# Enhancement of Antiviral Effect of Plastic Film against SARS-CoV-2: Combining Nanomaterials and Nanopatterns with Scalability for Mass Manufacturing

**DOI:** 10.1021/acs.nanolett.1c02266

**Published:** 2021-12-09

**Authors:** Yuyang Zhou, Nicola F. Fletcher, Nan Zhang, Jaythoon Hassan, Michael D. Gilchrist

**Affiliations:** † Centre of Micro/Nano Manufacturing Technology (MNMT-Dublin), School of Mechanical and Materials Engineering, University College Dublin, Dublin D04 KW52, Ireland; ‡ School of Veterinary Medicine, University College Dublin, Dublin D04 KW52, Ireland; § Conway Institute of Biomolecular and Biomedical Science, University College Dublin, Dublin D04 KW52, Ireland; ∥ National Virus Reference Laboratory, University College Dublin, Dublin D04 KW52, Ireland; ⊥ National Engineering Laboratory for Modern Silk, College of Textile and Clothing Engineering, Soochow University, Suzhou 215123, China

**Keywords:** COVID-19, nanostructure, film, ultrasonic atomization spray coating, thermal nanoimprinting lithography, scalable production

## Abstract

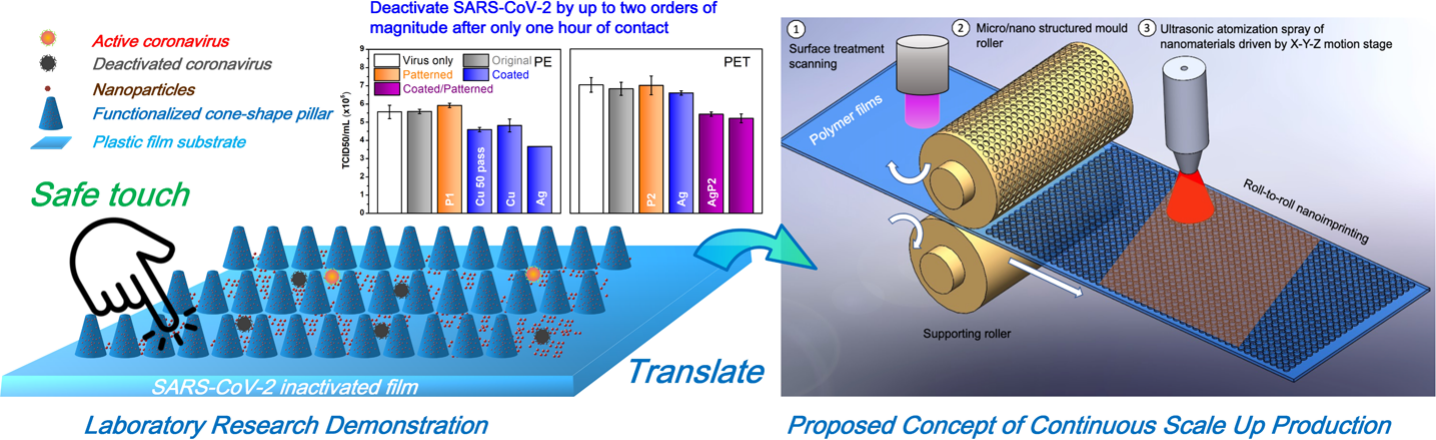

Direct contact with contaminated surfaces in frequently accessed areas is a confirmed transmission mode of SARS-CoV-2. To address this challenge, we have developed novel plastic films with enhanced effectiveness for deactivating the SARS-CoV-2 by means of nanomaterials combined with nanopatterns. Results prove that these functionalized films are able to deactivate SARS-CoV-2 by up to 2 orders of magnitude within the first hour compared to untreated films, thus reducing the likelihood of transmission. Nanopatterns can enhance the antiviral effectiveness by increasing the contact area between nanoparticles and virus. Significantly, the established process also considers the issue of scalability for mass manufacturing. A low-cost process for nanostructured antiviral films integrating ultrasonic atomization spray coating and thermal nanoimprinting lithography is proposed. A further in-depth investigation should consider the size, spacing, and shape of nanopillars, the type and concentration of nanoparticles, and the scale-up and integration of these processes with manufacturing for optimal antiviral effectiveness.

As of November 1 2021, the SARS-CoV-2 (COVID-19) pandemic has infected 246 million people and caused more than 5 million fatalities in more than 200 countries worldwide. Second, third, and fourth waves of the virus are still occurring around the world. With the continuing crisis, researchers are actively developing technologies to combat and cope with this and variant corona viruses.^1−5^ Thanks to the recent advent of several COVID-19 vaccines, efforts against the pandemic have now reached a historic turning point.^6^ However, living with the coronavirus into the future is increasingly likely, due to its pervasiveness and its continuing mutation. Thus, new innovations continue to be required to combat SARS-CoV-2.

Virus droplets larger than 5 μm diameter can travel up to 1 m. Smaller droplets can travel longer distances and upon contact with the nose, mouth, or upper respiratory tract, the airborne viral particles are inhaled by a person. Such a scenario is regarded as direct contact with infected individuals (i.e., not involving contaminated surfaces).^7−10^ Recommended preventive measures against direct contact include wearing a face mask covering in public, maintaining a minimum two-meter distance from other people, and monitoring and self-isolation for 14 days for people with a suspected infection. These measures are not always easy to implement and maintain but they successfully reduce the probability of direct transmission between humans. However, several studies have suggested that indirect contact transmission by touching a contaminated surface is the predominant transmission route for some respiratory viruses.^7,11^ Plastic products such as food packaging, handles, surfaces, door fixtures and commodity plastic products are widely used in all our daily lives. A number of control measures including adequate personal protective equipment (PPE) or antiviral surfaces for frequently touched objects are also being introduced gradually to high risk scenarios such as schools, health centers, and airports to reduce viral transmission. Recent research has confirmed that SARS-CoV-2 can remain active on plastic for 72 h, which poses a particularly high risk of indirect transmission by touching contaminated surfaces.^12,13^ For example, many outbreaks of coronavirus in food processing plants are due to cold and damp indoor areas, which are perfect environments for coronavirus to linger and spread.^14^ Virus-containing droplets from infected individuals are more likely to spread, settle, and stay viable. This has endangered workers and can also cause society transmission from frozen food packaging on which the coronavirus can survive longer. Moreover, the recent discovery of SARS-CoV-2 on the outer packaging of frozen foods is likely to be an important route for diseases transmission,^15^ and the use of protective antiviral film would mitigate this problem, which is particularly meaningful for international trade of frozen food products. Even though plastic surfaces can be disinfected using ethanol, hydrogen peroxide, or sodium hypochlorite, the process of sanitisation is laborious and requires the use of cleaning agents, can be difficult to achieve, and needs periodic repetition, which is not always feasible.^16^ Therefore, developing antiviral plastic surfaces that can be used widely and easily is required urgently in order to reduce the spread of viral infections in the population, and is in line with the requirement demonstrated in recent guidance documents from the European Centre for Disease Prevention and Control (ECDC).^17^

Significant progress has been made on the development of antimicrobial surfaces that prevent bacterial adhesion (Passive action) or actively kill bacteria that adhere to a polymer surface using active agents such as cationic biocides, antimicrobial peptides, or antibiotics (Active action).^18^ Recent research on micro/nano structured surfaces that mimic the topographical patterns of naturally occurring surfaces such as cicada and dragonfly wings, lotus leaves, and shark skin has also focused on antimicrobial functions.^19−21^ The main mechanisms concerning topography for bacterial attachment are physicochemical forces, cell membrane deformation, chemical gradients, hydrodynamics, surface wettability and air entrapment, topography-induced cell ordering and segregation, and the surface texture of a film.^22^ The advantages of a functional structured surface over a conventional coating combined with chemical regents are clear in terms of nontoxicity, high consistency, and low antimicrobial resistance.^23^ A very recent study demonstrates that a nanostructured aluminum (Al) alloy surface can deactivate SARS-CoV-2 by up to a 5 log reduction compared to a flat Al surface after 6 h of exposure.^24^ However, much less attention has been given to the practical issues of developing nanostructured antiviral polymer surfaces, specifically for use against the highly contagious coronavirus SARS-CoV-2. Because of the much smaller size of viruses, the diameters of which are usually between 20 and 300 nm with the exception of some few filoviruses which can be up to 1400 nm in diameter, the design principles that are used for antibacterial surfaces cannot be applied directly when developing antiviral surfaces or coatings.^25^ More significantly, the challenge at present is to translate laboratory research achievements to scalable production in order to ensure that such benefits can rapidly have a positive impact on public health.

Achieving micro/nano structures on polymer surfaces involves multidisciplinary and interdisciplinary science and technologies, including biomimicry, material science, bioscience, and advanced manufacturing.^23,26^ The specific challenge concerns how to replicate micro/nano structures on frequently used flexible plastic films rather than on hard and rigid bulk substrates such as stainless steel, metals, and silicon; this is challenging due to the softness, flexibility, and thinness of such films. Currently, there are three main techniques for patterning surfaces, namely, lithographic techniques, direct writing techniques, and instability-induced polymeric patterning to achieve either orderly or random nanopatterns.^27^ However, orderly nanopatterning requires high precision manufacturing technology, such as direct laser writing or electron-beam lithography, which inhibits scalable production due to their high manufacturing cost. Nanoimprinting lithography (NIL) is a low-cost nanopatterning method that offers high throughput and high resolution, especially for producing large-area micro/nano scale patterns with high-aspect-ratio features.^28^ The random patterned anodized aluminum oxide (AAO) template is easily accessible through a chemical anoidization process and can be easily integrated with NIL processes. As yet, however, relatively little research has incorporated using NIL technology for scalable replication of nanostructured plastic films (e.g., PE or PET) against SARS-CoV-2. The manufacturing process and the antiviral efficacy of structural plastic films have yet to be properly demonstrated.

It has long been recognized that silver nanoparticles (AgNPs) are effective antiviral agents for preventing infections and resisting putrefaction of food and are less likely to develop resistance compared to conventional antivirals.^29^ AgNPs are able to combat a wide range of viruses including retroviruses (i.e., HIV virus), herpesviruses, paramyxoviruses (such as Respiratory Syncytial Virus), Hepatitis B virus, and influenza virus.^29−31^ Specifically, AgNPs have been shown to bind preferentially to viral surface proteins rich in sulfhydryl groups and cleave the disulfide bonds, which destabilizes the protein, thereby affecting viral infectivity; they also possess intracellular antiviral action by interacting with viral nucleic acids.^32,33^ Importantly, AgNPs have been demonstrated to be nontoxic to humans and can be dispersed easily or coated onto a variety of surfaces, such as wound dressings, mask filtering layers, computer keyboards, elevator handrails, chairs and interior trim in trains, coaches, subways, and so forth. Similarly, copper destroys the replication and propagation abilities of SARS-CoV, influenza virus, HIV, and fungi after a short period of exposure.^34^ Copper has long been regarded as a safe antiviral reagent as it is one of the useful nutrients required for normal functioning of the human body and is also an effective and low-cost complementary strategy to help reduce the transmission of several infectious diseases by limiting transmission of nosocomial infections.^34,35^ The antiviral activity of copper nanoparticles (CuNPs) is mainly a contact mechanism through metal ion binding, which is especially effective on the enveloped virus.^36,37^ To date, the effectiveness of AgNPs and CuNPs against SARS-CoV is widely recognized and has been proposed for medicinal applications.^38,39^ Much recent progress has been achieved on the surface of stainless steel using Ag coatings and on facial masks using Cu compounds and copper–silver (Cu–Ag) nanohybrids against SARS-CoV-2.^40−42^ However, demonstration and use of NPs as antiviral coatings on plastic surfaces remains rare. There are three major strategies to achieve antimicrobial polymers, namely, (a) incorporate antimicrobial agents directly into polymers during plastic processing, (b) coat or immobilize antimicrobials onto polymer surfaces, and (c) use polymers that are inherently antimicrobial.^43^ Polymer surface coatings and the fixing of antimicrobials are technologies that are widely used, cost-effective, and have less of a negative impact on the intrinsic nature of the substrate. To date, antiviral surfaces are mainly achieved by means of natural antiviral coatings, physically/chemically modified antiviral coatings, and bioinspired antiviral surfaces based on various technologies, such as solution and dip coatings, cast-coating, spraying methods, and spin coating.^44−46^

Consequently, in the present paper we propose a novel design for polymer films that efficiently deactivate SARS-CoV-2 by means of a uniform and relatively durable NP coating that is combined with nanoscale conical pillars in order to minimize the transmission route via plastic film surfaces among people (Figure 1). The nanostructural design aims to increase the effective contact area between SARS-CoV-2 and the nanocoatings. Our approach is particularly suitable for scalability and is compatible with commercial manufacturing process technology that would be required for the production of antiviral films. Doing so would require only integrating ultrasonic atomization spray coating (UASC) for uniform coatings and thermal nanoimprinting lithography (TNIL) for the replication of large-area micro/nano scale and high-aspect-ratio patterns with existing production processes.

**Figure 1:**
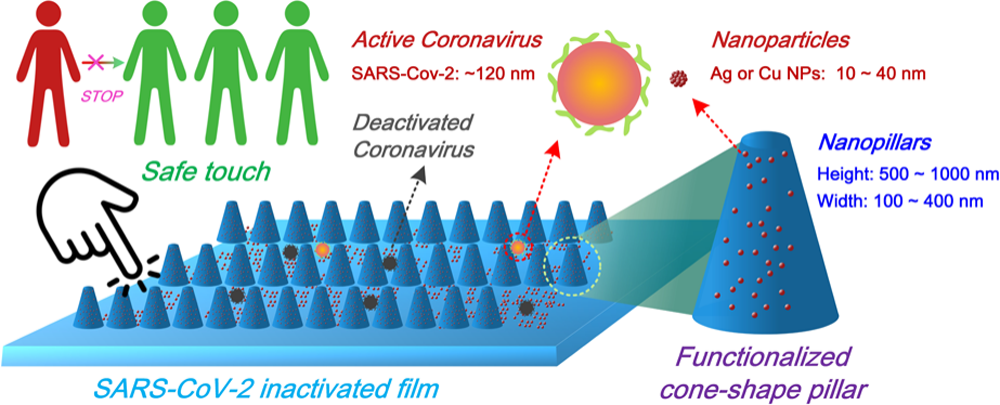
Schematic for antiviral micro/nano structural surface design.

We first focused on the ink formulation for uniform and short-term durable NP surface coatings. Surface tension, viscosity, and particle size distributions are the main parameters that need to be optimized. Besides using the effective antiviral components (AgNPs and CuNPs, the size of which vary between 10 and 40 nm), we also introduced sodium dodecyl sulfate (SDS) and polyvinyl acetate (PVA) to enable easier spreading of the atomized droplets during the UASC process and the enhanced adhesion of NPs, respectively. The most challenging issue we addressed during ink formulation was determining an appropriate PVA concentration. We found that too high a PVA concentration in the ink formulation could block the tiny spraying nozzle due to its high viscosity, thus disabling the UASC process (Figure S1) and also fully submerge the NPs and pillars which would cause poor contact with the virus (Figure S2). At the other extreme, too low of a concentration of PVA fails in its role as an adhesive (Figure S3). With the introduction of SDS and PVA (optimal concentrations: SDS, 2 g/L; PVA, 1 mg/L), the surface tension of the ink decreased by ∼20% and its viscosity increased by ∼5% for both AgNPs and CuNPs ink formulations compared with the respective original suspensions (Figure 2a). The addition of SDS and PVA had only a marginal impact on the particle size and distribution (curve shape and polydisperse index (PDI)) (Figure 2b). The slight increase of the NPs size is probably due to the PVA adhesion between the NPs. The resultant ink was used subsequently in the UASC process.

**Figure 2:**
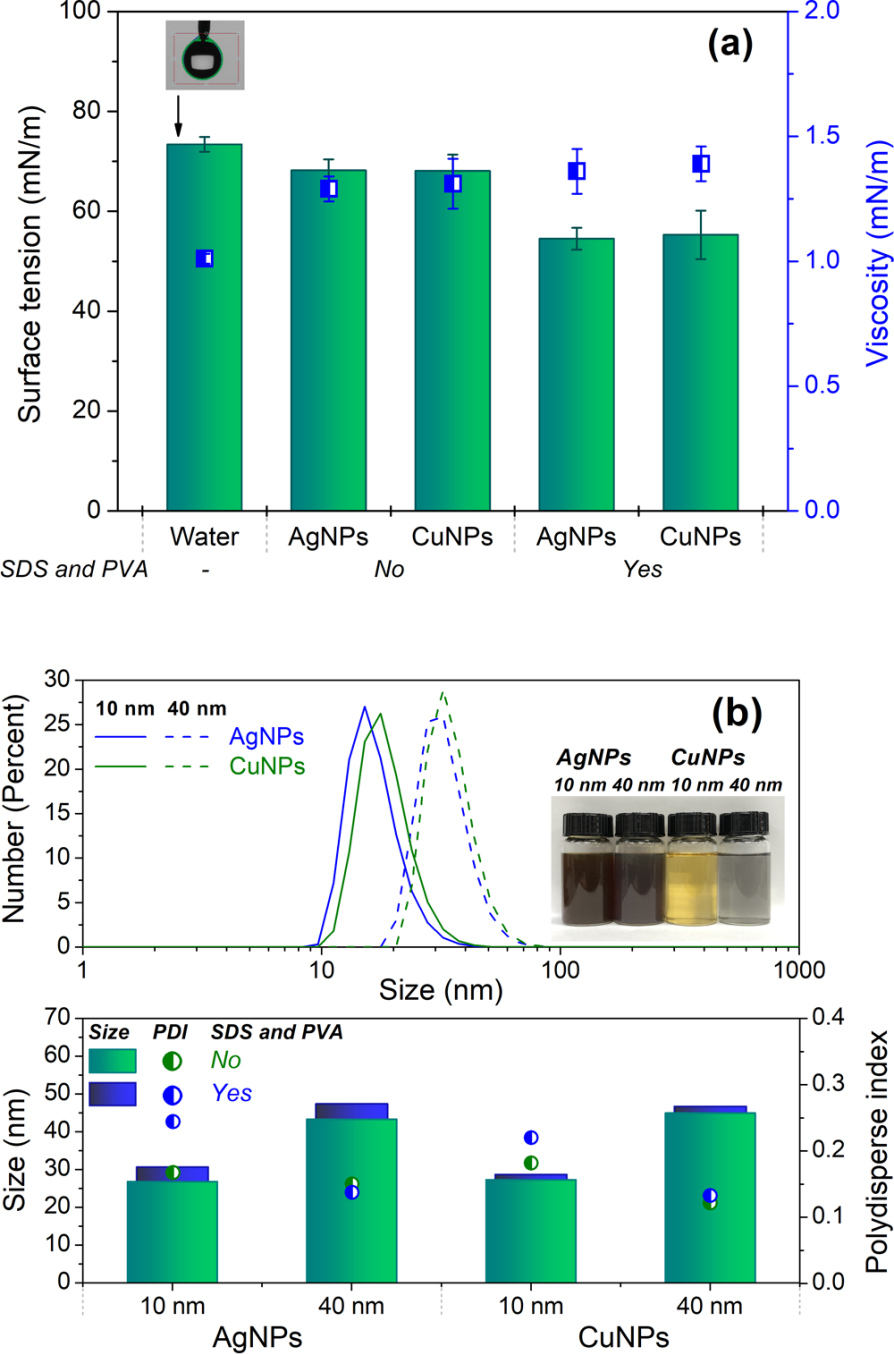
Ink formulation for UASC (a) surface tension and viscosity; (b) particle size and distribution.

Decreasing the contact angle (CA) of the film surface facilitates the uniform spreading of tiny droplets during the UASC process (Figure S1). Moreover, lowering the CA of the final film also enhances the contact area of viral droplets with the antiviral surface coating. Polyethylene terephthalate (PET) and polyethylene (PE) films were used as representative substrates with widespread industrial relevance for surface treatment and antiviral assessment. Original (i.e., untreated) PE and PET films displayed CA values of 84.2° and 76.7°, respectively (Figure 3a). After the ultraviolet (UV) ozone treatment, the CA decreased to 60–70° due to the temporary introduction of hydrophilic functional groups such as −OH, −CHO, and −COOH.^47,48^ Afterward, the pretreated films were directly subjected to the strip-by-strip UASC process. The TNIL (Figure S4) using two conical porous anode aluminum oxide (AAO) templates had no significant effect on the CA of the films. A larger micro/nano structure (pillar height of P2, ∼1000 nm) contributed to a slightly greater decrease in CA than the small one (pillar height of P1, ∼400 nm). On the other hand, there was a decrease in CA after the UASC process; this was mainly due to the hydrophilic coating containing SDS. Both the UASC and TNIL processes reduced the transparency of the films marginally but not sufficiently to render them unsuitable for practical applications such as packaging films. Films coated with AgNPs displayed a slight yellowish appearance due to the dark brownish ink formulation (see inset photo in Figure 2b). Films such as packaging films are unavoidably subject to hand touching during usage. To evaluate the abrasion durability, the sample films were examined after five cycles of abrasion under 1 N loading on a pin-on-disk wear test machine with a customized rubber pin to simulate repeated heavy handling situations (Figure S5). The durability of coatings was significantly enhanced by using the PVA. No obvious scratches or wear were observed on the coatings after these wear tests (Figure 3c). The integrity of the nanostructures also remained intact on the film surfaces (Figure S6). The morphology of coated and patterned films was examined further using both a 3D surface metrology system and scanning electron microscopy (SEM) (Figure 3d). Obvious conical pillars and NPs were observed clearly on both the PET and PE films, demonstrating the successful spray coating and nanopatterning processes. The larger contact area of the surface structure contributes to the higher deactivation efficiency against SARS-CoV-2. Thanks to the UV ozone treatment followed by the UASC process using hydrophilic ink, the droplets are distributed uniformly on the film surface after only a few seconds (See SI Video 1). Moreover, PET film was more easily patterned than PE film; this is likely due to its higher *T*_g_, enabling easier demolding under ambient processing conditions.

**Figure 3:**
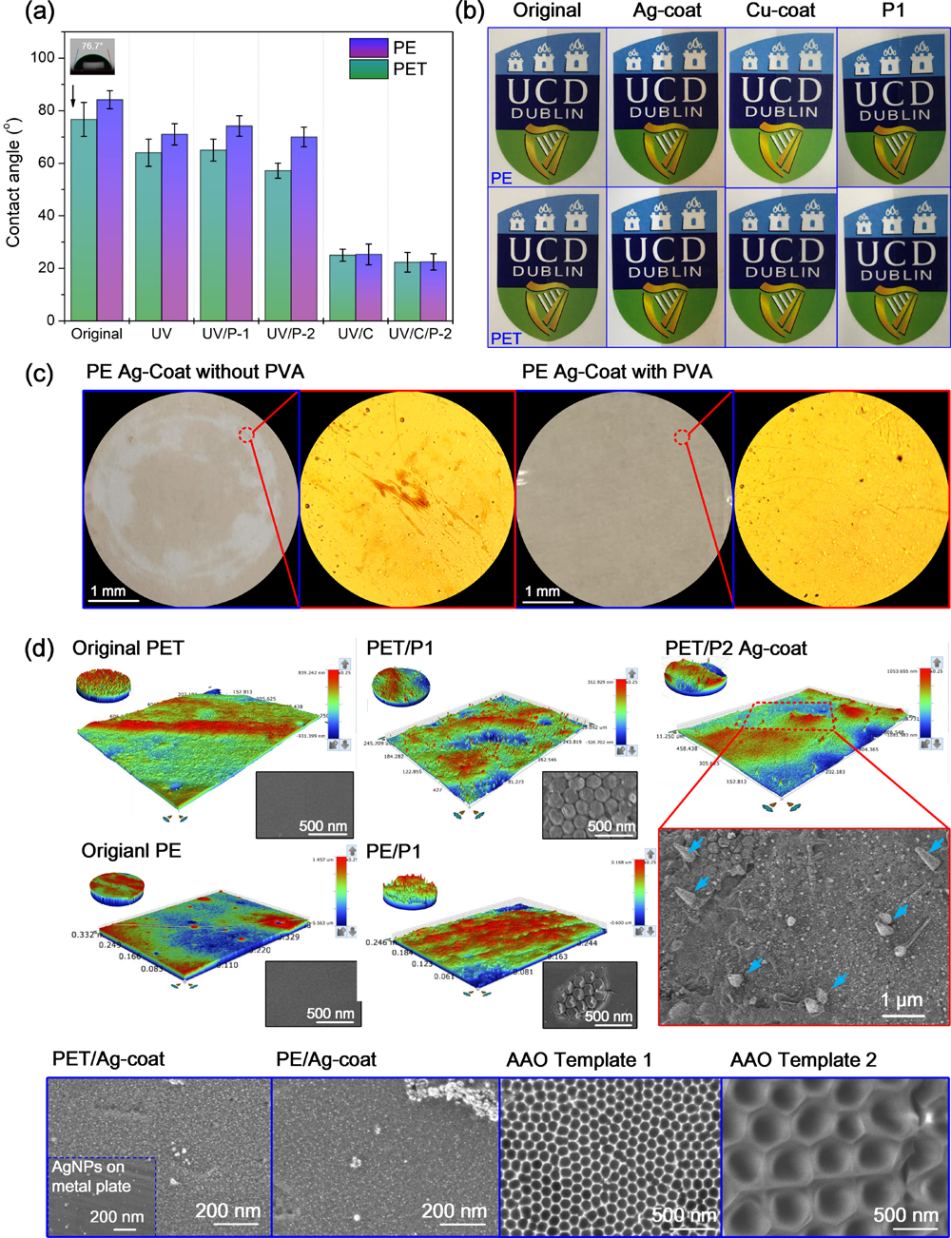
Film characterizations of the following: (a) contact angle; (b) transparency; (c) wear durability; (d) morphology images by 3D surface metrology system and SEM. Note: The images of UCD crest were permitted for use by University College Dublin.

Antiviral assessments were carried out on 15 film samples through 50% Tissue Culture Infective Dose (TCID50). Three batches were characterized separately with an untreated virus control included in each experiment (Figure 4). Original (i.e., untreated) PE and PET films (surfaces (2) and (8), respectively, as indicated in Figure 4) showed no antiviral effectiveness. Patterns without the use of either AgNPs or CuNPs also displayed no impact on SARS-CoV-2. Patterned PE (surfaces (3) and (14)) showed slightly higher antiviral activity against SARS-CoV-2 than patterned PET (surfaces (9) and (16)). This may be due to the slightly higher hydrophilicity of PET than PE film, leading to a higher area of contact with the viral droplet, which was observed during SARS-CoV-2 assessment. The contact angles were also confirmed in Figure 2. Upon the coating of NPs on the film, significant deactivation of SARS-CoV-2 was observed after only 1 h of viral contact. At the same concentration, AgNPs (surface (6)) were found to be more effective than CuNPs (surface (5)), even though the number of spray passes increased to 50 (surface (4)). The efficient antiviral activity of AgNPs is due to interference with cellular receptor binding, inhibition of viral replication, and viral inhabitation by Ag^+^ ions released from AgNPs.^30^ A concentration of 100 ppm of AgNPs imparted the PET film with a low antiviral effect (surface (10)). However, the combination of AgNPs coating and nanopatterning (surface (11)) significantly enhanced the antiviral effect compared with coating only (surface (10)). This positive effect confirmed our preliminary assumption, that is, increasing the contact area through the use of nanopatterns enhances antiviral efficiency. There was no obvious further reduction in viral infectivity when the size of AgNPs was increased to 40 nm at a concentration of 300 ppm (surface (12)), which indirectly implied the higher antiviral efficiency of smaller AgNPs. This phenomenon is consistent with previous studies. In fact, there are three major factors that influence the antiviral activity of NPs, namely, the particle size, concentration, and contact time. Larger-sized AgNPs could influence agglomeration and rate of dissolution properties of the AgNPs, thus resulting in lower antiviral activity.^33,49,50^ Increasing the concentration of AgNPs before TNIL can further improve the antiviral effectiveness (surfaces (17) and (18)). There was no significant difference between the type of films (PE or PET) (surfaces (15) and (18)) after the UASC and TNIL processes, which implies that our developed strategy could potentially be used to functionalize other types of plastic films. In general, a 2 log reduction in viral infectivity was observed after 1 h of contact with the functionalized films using our designed protocol. This corresponds to 2 orders of magnitude or a 100-fold reduction in infectivity. Our preliminary studies considered longer contact times than 1 h in which we observed further reductions in the levels of infectivity (data not shown).

**Figure 4:**
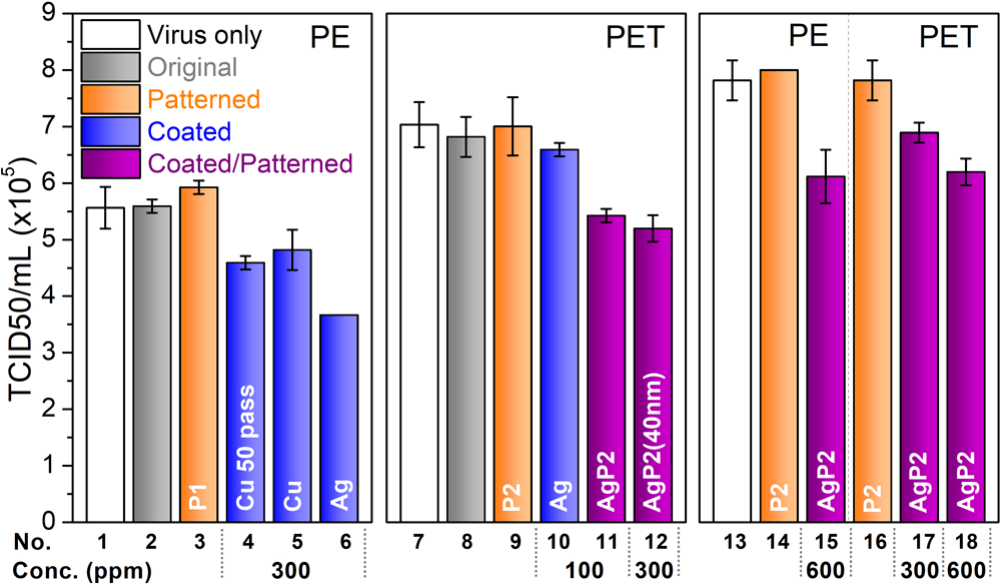
Antiviral assessment of functionalized films. Note: Patterns (P1, 125-040-250; P2, 450-100-1500). Spray coatings were carried out for 20 passes except for surface (4), which was sprayed for 50 passes. Diameter of NPs were 10 nm except for surface (12) for which they were 40 nm.

The current process developed to fabricate antivirus films shows high potential for scalability at relatively little cost or complexity, as proposed in Figure 5. Such films are conventionally fabricated by an extrusion process, after which a roll of plastic film can be used for roll-to-roll nanoimprinting. Surface activation, by means of UV or plasma treatment, can be integrated into nanoimprinting systems to enhance surface wettability. Meanwhile, surface micro/nano structural topography optimization can also be used to tune the surface wettability of polymers. Along with formulating nanomaterial inks, an optimized spray coating of nanomaterials with uniform thickness could be achieved. Atomisation spray of nanomaterials could be integrated easily into film production processes by means of an automated controller using an X-Y-Z stage. A multinozzle system could be used to deposit multilayer materials or to enhance production efficiency. Finally, films can then be rolled and supplied for fabrication of antivirus and antimicrobial products, such as commodity packaging, protective shields, and handle and elevator covers, that could be used in public areas such as buses and public transportation, schools and supermarkets, and so forth. It is worth noting that successful process integration would involve overcoming various technical challenges and would also require iterative optimization. For example, fabrication of the roll mold would require developing an AAO-based roller mold which could be fabricated by means of anodization or a nickel-based nanomold which could be fabricated using electron beam lithography and electroforming.^51,52^ The nanoimprinting process based on roll-to-roll thermal nanoimprinting, or the roll-to-roll extrusion coating nanoimprinting and coating process, would also need to be optimized in order to achieve good replication fidelity of the designed nanopattern and uniformity of the coating.^53,54^ Such processes have been demonstrated in industry for fabricating functional nanopatterns such as antireflection, superhydrophobic, and nanophotonic surfaces.^55−57^ There are likely to be additional challenges with coatings. The UASC process could be integrated into a production line by using multiple ultrasonic spraying nozzles. However, it might need a high level of ventilation and protection for the spraying process. Alternatively, a roll coating process could be used to match the production speeds of nanostructured films that are as fast as 60 m/min. Such coating processes have been used in industry to coat adhesive materials or photoresists to polymer carrier foils. The surface wettability of a film material could be changed by plasma activation so that the coating can be applied to nanostructured polymer surfaces, where the coating formulation needs to be optimized. A brief cost assessment for scalable production of antiviral polylactic acid (PLA) film can be achieved by calculating the consumption of materials and manufacturing consumables for fabrication of each square meter. PLA pellets cost ∼40 Euro/kg, thus 1 m^2^ of PLA film with a thickness of 0.1 mm is estimated to be 4.96 Euro. Around 100 mL of nanoparticles would cost 0.70 Euro (Nanoparticles-300 ppm-7 Euro/L) for 1 m^2^. With respect to the manufacturing set up, a roller mold (c. 15 000 Euro), an ultrasonic spray head and generator (c. 3350 Euro) are expected to run continuously to produce 100 000 m^2^ of PLA film. Thus, each square meter costs 0.18 Euro. In general, one square meter of functionalized PLA film costs 5.84 Euro, which is merely 0.88 Euro more than normal PLA film (i.e., an extra 18%) but having antiviral capability based on our proposed processes. Clearly, this is quite cost-effective.

**Figure 5:**
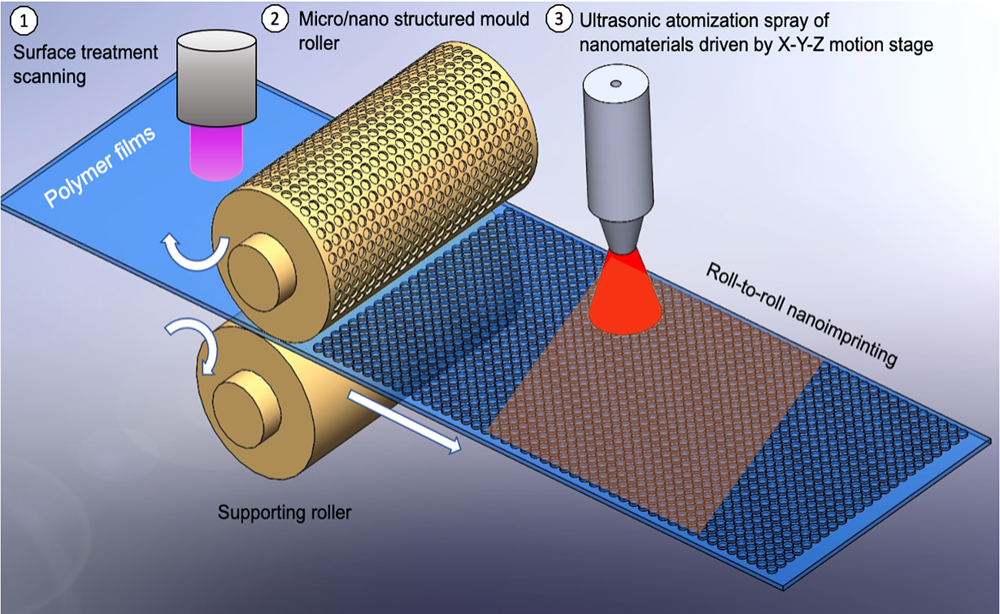
Proposed scale up production of antimicrobial and antivirus film. Surface activation of polymer films via UV or plasma treatment to enhance wettability, and the use of a micro/nano structured mold roller to create surface texture that would enhance wettability and increase the contact area between virus and nanomaterials though roll-to-roll nanoimprinting process; the ultrasonic atomization process is integrated to deposit functional nanoinks for antivirus and antimicrobial surfaces.

Overall, we believe that this proposed process is appropriately scalable and compatible with exisiting industrial low cost production processes and thus has strong commercialization potential. We are separately intending to develop biobased materials for antivirus/antimicrobial films using the scientific and technical achievements of this present research, and we intend to expand the applicable range of products to include the food and medical packaging industries based on this proposed scale-up strategy.

## Experimental Section

### Antiviral Ink Formulation

Antiviral ink for ultrasonic atomization spray coating was prepared by mixing 100–600 ppm AgNPs or CuNPs suspensions (Guangzhou Hongwu Materials Technology Co., Ltd.) with 2 g/L SDS (Sigma-Aldrich) and 1 mg/L PVA (Sigma-Aldrich). The mixture was magnetically stirred for 5 min followed by high-power ultrasonic-assisted dispersing for 10 min using a LULP500 Ultrasonic Liquid processor (Cheersonic Ultrasonic Co.). The as-prepared ink was loaded in a glass syringe.

### UASC

UASC was carried out on a customized surface coating workstation equipped with UAC120 ultrasonic atomizer system (Cheersonic Ultrasonic Co.). Twelve micron thick polyethylene terephthalate (PET) and 50 μm thick polyethylene (PE) films were kindly provided by Foxpak Flexibles Ltd. Before UASC, films were rinsed with acetone and pretreated under a UV ozone cleaner (Ossila Ltd.) for 30 min. UASC was implemented strip by strip at an infusion rate of 0.5 mL/min under 0.01 MPa gas pressure (see SI Video 2) with tiny droplets of micrometer level diameter (Figure S1). The resultant films were dried in open air.

### TNIL

TNIL process was carried out on a Nanoimprint Tool (NIL Technology, Denmark) under pressure of 5 bar at temperatures of 85 °C for PE and 180 °C for PET. The sample loading in the TNIL equipment is illustrated schematically in Figure S4. Two conical porous anode aluminum oxide (AAO) templates with different specifications (pore diameter at the top - pore diameter at the bottom - pore depth (nm): Template 1, 125-40-250; Template 2, 450-100-1500) were used. AAO templates were fluorinated with trichloro (1H, 1H, 2H, 2H-perfluoroctyl) (Sigma) via a thermal vapor and deposition process before TNIL.

### Characterizations

Viscosity of the ink was measured on a DV3T rheometer (AMETEK Brookfield, U.S.A.). Contact angle (CA) and surface tension of the ink were tested in a contact angle goniometer (Ossila, U.K.). The films were observed using an SP-99 FL microscope equipped with a high-resolution camera (Brunel, U.K.). The morphologies of films were measured with NPFLEX 3D Metrology System (Bruker, U.S.A.) and Hitachi Quanta 3D FEG SEM (Hillsboro, U.S.A.).

Abrasion testing was carried out on a Pin-on-disk Friction Wear Test system (NEO-Tribo MFW120, Korea). The test film was stabilized horizontally on the sample stage using adhesive tape (Figure S5). A wear pin with a 2 mm diameter area of nitrile rubber end was custom designed to simulate a manual handling process. The machine was run at a speed of 10 rpm for five cycles using a rotational mode at a radius of 4 mm under a 1 N loading. The pillars remained unchanged after abrasion testing (Figure S6).

### Cell Culture

VeroE6 cells (ATCC CRL-1586) were cultured in Dulbecco’s Modified Eagle’s Medium (DMEM) supplemented with 2% fetal bovine serum (FBS).

### Antiviral Evaluation Experiments

Antiviral evaluation of prepared films was carried out at the BSL-3 containment facility at the Veterinary Sciences Centre, University College Dublin, using 50% Tissue Culture Infectious Dose (TCID50) assays. Fifty microliters of SARS-CoV-2 (2019-nCoV/Italy-INMI1 from EVA global) was added to the surface of each plastic film for 1 h at room temperature. Control, untreated virus was stored at room temperature for 1 h within a sterile screw cap vial. The virus was then titrated in 1:10 serial dilutions in quadruplicate on VeroE6 cells and incubated at 37 °C for 72 h. VeroE6 cells were scored for cytopathic effect (CPE) (+ or −) and TCID50 calculated according to the method of Reed and Muench.^58^

## Supporting Information Available

Movie of CA tests on original film and antiviral ink-coated PE film (MP4)
Movie of ultrasonic atomization spray coating (MP4)
Photos for tiny droplets generated during UASC; SEM image of PE film after UASC; Photo of PE film after UASC after wear test; A scheme for TNIL equipment; Wear test setup with a customized rubber pin; 3D morphology of nanopatterned film after wear test (PDF)
