# Supplementary material for: Enhancement of Antiviral Effect of Plastic Film against SARS-CoV-2: Combining Nanomaterials and Nanopatterns with Scalability for Mass Manufacturing
Source: Nano Lett. 2021 Dec 9;21(24):10149–56. doi: 10.1021/acs.nanolett.1c02266 (PMC8672428; doi:10.1021/acs.nanolett.1c02266)
Supplement: Supplementary file 3 — nl1c02266_si_003.pdf [file nl1c02266_si_003.pdf]

Supporting Information

Enhancement of antiviral effect of plastic film against SARS-CoV-2: Combining nanomaterials and nanopatterns with scalability for mass manufacturing

Yuyang Zhou[1,5], Nicola F. Fletcher[2,3], Nan Zhang[1,*], Jaythoon Hassan[4], Michael D. Gilchrist[1]

[1] Centre of Micro/Nano Manufacturing Technology (MNMT-Dublin), School of Mechanical & Materials Engineering, University College Dublin, Dublin, D04 KW52, Ireland

[2] School of Veterinary Medicine, University College Dublin, Dublin, D04 KW52, Ireland

[3] Conway Institute of Biomolecular and Biomedical Science, University College Dublin, Dublin, D04 KW52, Ireland

[4] National Virus Reference Laboratory, University College Dublin, Dublin, D04 KW52, Ireland

[5] National Engineering Laboratory for Modern Silk, College of Textile and Clothing Engineering, Soochow University, Suzhou, 215123, China

* *Corresponding author:*

nan.zhang@ucd.ie (N. Zhang)

Content **Page**

6 figures in 3 pages

Fig. S1-S3 *S2*

Fig. S4-S6 *S3*

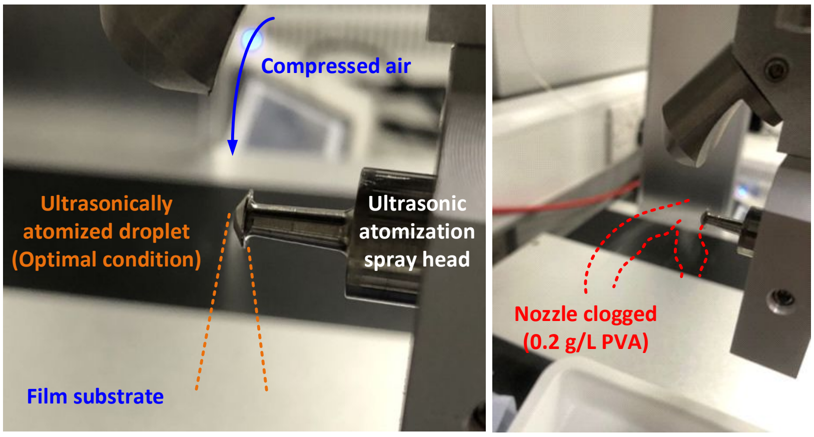

Fig. S1 Photos for tiny droplets generated during UASC.

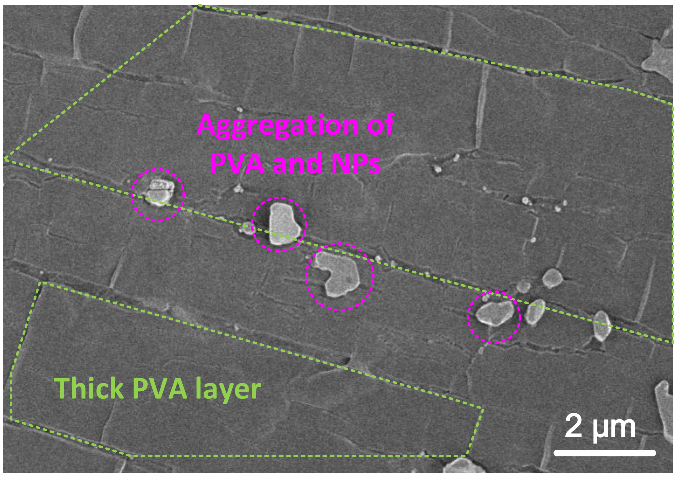

Fig. S2 SEM image of PE film after UASC (300 ppm AgNPs, 0.2 g/L PVA, 2 g/L SDS, 10 passes).

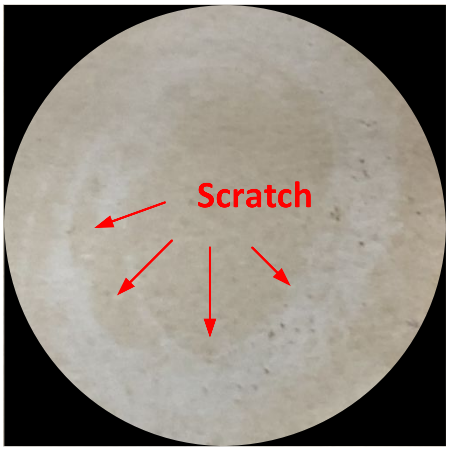

Fig. S3 Photo of PE film after UASC (300 ppm AgNPs, 0.1 mg/L PVA, 2 g/L SDS, 20 passes) after wear test.

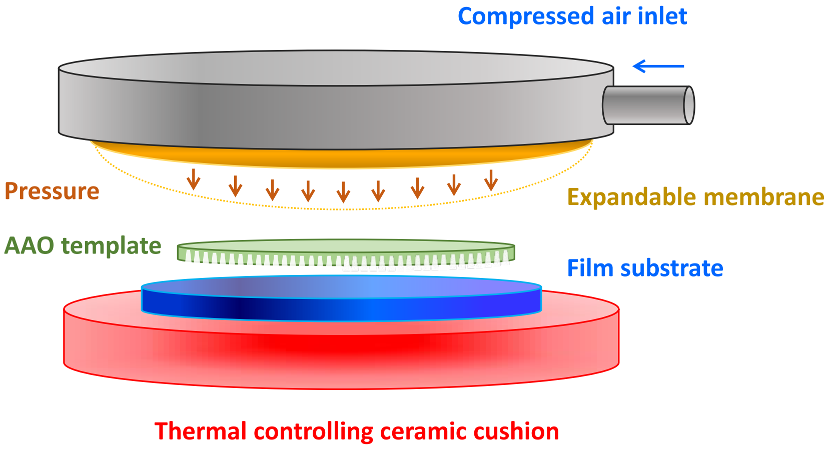

Fig. S4 A scheme for TNIL equipment.

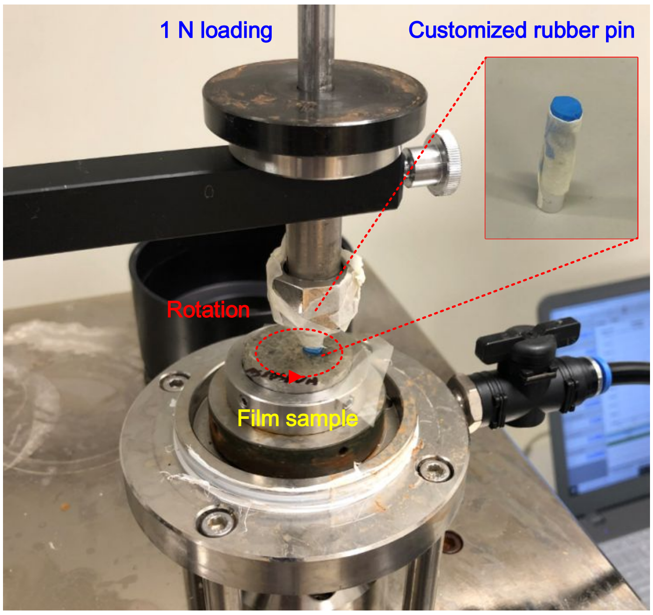

Fig. S5 Wear test setup with a customized rubber pin.

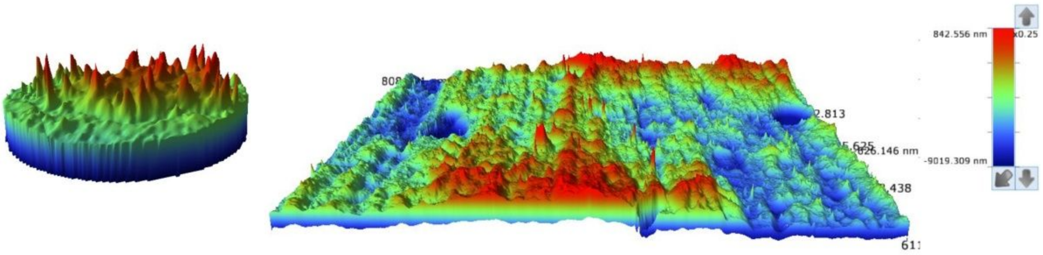

Fig. S6 3D morphology of nanopatterned film after wear test.
